# Increased risk of pancreatic cancer in individuals with non-alcoholic fatty liver disease

**DOI:** 10.1038/s41598-022-14856-w

**Published:** 2022-06-23

**Authors:** Joo-Hyun Park, Jung Yong Hong, Kyungdo Han, Wonseok Kang, Joo Kyung Park

**Affiliations:** 1 Department of Family Medicine, Korea University Ansan Hospital, Korea University College of Medicine, Ansan, Korea; 2 Department of Healthcare Administration and Policy, School of Public Health, University of Nevada Las Vegas, Las Vegas, USA; 3 Division of Hematology-Oncology, Department of Medicine, Samsung Medical Center, Sungkyunkwan University School of Medicine, Seoul, Korea; 4 Department of Statistics and Actuarial Science, Soongsil University, Seoul, Korea; 5 Division of Gastroenterology, Department of Medicine, Samsung Medical Center, Sungkyunkwan University School of Medicine, Seoul, Korea; 6 Department of Health Sciences and Technology, SAIHST, Sungkyunkwan University, Seoul, Korea

**Keywords:** Cancer prevention, Oncology, Risk factors

## Abstract

The association between non-alcoholic fatty liver disease (NAFLD) and the risk of pancreatic cancer in the general population remains unclear. This nationwide cohort study included 8,120,674 adults who underwent a national health screening in 2009 from the Korean National Health Insurance Service database. Participants were followed-up until December 2017 for the development of pancreatic cancer. NAFLD was assessed using the fatty liver index: ≥ 60, NAFLD and < 30, no NAFLD. Multivariable Cox proportional hazards regression was performed. During the follow-up of 59.1 million person-years, 10,470 participants were newly diagnosed with pancreatic cancer. NAFLD was significantly associated with an increased risk of pancreatic cancer compared to no NAFLD (adjusted hazard ratio [aHR], 1.17; 95% CI 1.09–1.26). This association was significant in both the obese (aHR, 1.14; 95% CI 1.05–1.23) and non-obese groups (aHR, 1.14; 95% CI 1.003–1.29). Individuals with fatty liver index 30–59 also had an increased risk (aHR, 1.10; 95% CI 1.05–1.16). The risk of pancreatic cancer increased with increasing fatty liver index scores (*P* for trend < 0.001). This study demonstrated that NAFLD was independently associated with an increased risk of pancreatic cancer, regardless of obesity. Our finding suggests that NAFLD may be a modifiable risk factor for pancreatic cancer.

## Introduction

Pancreatic cancer is one of the most lethal diseases, with an overall 5-year survival rate of approximately 9%^1^. Approximately 60,000 new cases of pancreatic cancer are diagnosed annually in the United States^2^. The incidence of pancreatic cancer is increasing, and it is predicted to become the second leading cause of cancer-related deaths by 2030^3,4^. However, evidence supporting the utility of pancreatic cancer screening is still lacking^5^. Thus, identifying modifiable risk factors for pancreatic cancer should be prioritized to reduce the burden of pancreatic cancer worldwide^1,3,5^.

Non-alcoholic fatty liver disease (NAFLD) refers to excessive fat accumulation in the liver with no heavy alcohol consumption or other secondary causes of steatosis^6^. The incidence and prevalence of NAFLD are rapidly increasing worldwide^7^. Recent evidence has revealed that NAFLD is a risk factor for several cancers, such as hepatocellular carcinoma^8–10^ and colorectal cancer^10^.

However, the association between NAFLD and the risk of pancreatic cancer remains unclear. Only a few small hospital-based studies were conducted, and these studies showed inconsistent results of positive^11–14^ and null associations^15^. Moreover, these studies did not adjust for known risk factors related to pancreatic cancer such as pancreatitis^11–15^, body mass index (BMI)^11–15^, and smoking^11,12,14^. Notably, no study has assessed the effect of NAFLD on the risk of pancreatic cancer in the general population.

To assess NAFLD, liver biopsy; imaging studies; and non-invasive biomarkers, including the fatty liver index, have been used^10,16–21^. However, performing a liver biopsy or imaging studies is not feasible in the asymptomatic general population. As recommended by the recent international guidelines from the European Association for the Study of the Liver, non-invasive serum biomarkers are the preferred assessment tool for NAFLD in large-scale population-based studies^10^.

Therefore, we conducted this nationwide, population-based cohort study of over 8 million adults to investigate the association between NAFLD and pancreatic cancer risk using the fatty liver index in the general Korean population.

## Materials and methods

### Data source

We used the data from the national health screenings and the Korean National Health Insurance Service (KNHIS), which is a mandatory national health insurance system managed by the government. The KNHIS covers approximately 97% of the total population. The remaining 3% of the population is covered by the Medical Aid Program, where their claims data are also reviewed by the KNHIS. Therefore, the KNHIS database covers the entire Korean population.

The KNHIS provides a standardized biannual national health screening program for citizens ≥ 20 years of age. The national health screening data includes anthropometric measurements, laboratory test findings, past medical history, and health-related behaviors. Using the KNHIS database, we obtained clinical information, including demographics, diagnostic codes, medical treatment-related data (prescriptions, hospital admissions, and procedures), and results of the national health screenings^22^. The KNHIS claims database uses the International Classification of Diseases-10th Revision-Clinical Modification (ICD-10-CM) codes.

In 2006, the KNHIS also performed a registration program with special reimbursement codes to lower the copayment rate to 5% for cancers (V codes). All patients with such diseases are required to have their diagnosis certified by a physician to receive the payment benefits for cancer-related management. Thus, V codes based on national registration data for cancer patients are reliable.

The present study was approved by the Institutional Review Board of the Samsung Medical Center (#SMC2019-08-106) and the KNHIS Big Data Steering Department (NHIS-2019-1-499) and was exempted from informed consent requirements. The study was conducted in accordance with the principles of the 1964 Declaration of Helsinki. The KNHIS data were obtained from the Korea National Health Insurance Sharing Service after receiving permission from the Institutional Data Access/Ethics Committee.

### Study population

**Figure 1:**
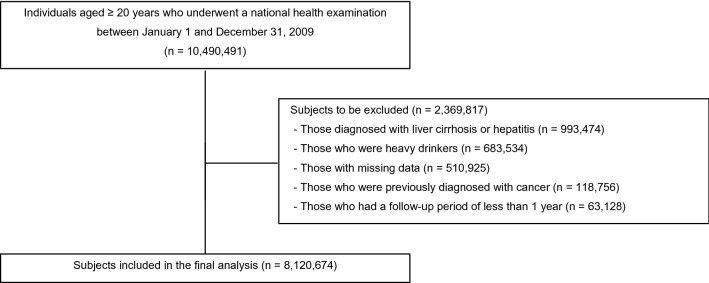
Flow diagram for the selection of the study population.

Figure 1 shows the selection process of the study population. We included 10,490,491 adults aged ≥ 20 years who underwent a health examination provided by the KNHIS between January 1 and December 31, 2009. To define NAFLD, we excluded patients with alcoholic liver cirrhosis (ICD-10-CM Code K703; *n* = 81,916), patients with hepatitis (ICD-10-CM code K746; *n* = 911,558), and those with heavy alcohol consumption (≥ 30 g of alcohol per occasion; *n* = 683,534)^23^. We excluded individuals with a previous diagnosis of cancer (*n* = 118,756). To avoid potential reverse causality, we further excluded participants who developed pancreatic cancer or died within the first year of entering the cohort (*n* = 63,128). We also excluded participants with missing variables (*n* = 510,925). Finally, 8,120,674 participants were included in the study and followed-up until the date of pancreatic cancer development, death, or December 31, 2017, whichever came first.

### Clinical variables and biochemical analysis

A trained clinician measured the patients’ height, weight, and waist circumference. The BMI was calculated by dividing the weight by height squared (kg/m^2^). Obesity was defined as a BMI ≥ 25 kg/m^2^ according to Asian standards^24^.

Blood samples were obtained after overnight fasting to measure the serum levels of glucose, low-density lipoprotein cholesterol, high-density lipoprotein cholesterol, triglycerides, total cholesterol, γ-glutamyl transferase (GGT), alanine aminotransferase, and aspartate aminotransferase.

Lifestyle-associated covariates, including smoking history (none, ex-smoker, or current smoker), alcohol consumption (none, mild consumption [< 30 g of alcohol per day], heavy alcohol consumption [≥ 30 g of alcohol per day])^25^, and physical activity (high-intensity activity ≥ three times/week or moderate-intensity activity ≥ five times/week), were also evaluated using standardized self-administered questionnaires. Income level was dichotomized into the lowest quartile (25%).

Comorbidities were defined as follows: pancreatitis (ICD-10-CM codes K85, K86.0, and K86.1); diabetes mellitus (fasting blood glucose levels ≥ 126 mg/dL measured during the health screening or ≥ one claim per year for ICD-10-CM codes E10–E14 and a prescription for an antidiabetic drug); dyslipidemia (fasting blood total cholesterol levels ≥ 200 mg/dL measured during the health screening or ≥ one claim per year for ICD-10 Code E78 and a prescription for lipid-lowering medications); and hypertension (systolic/diastolic blood pressure ≥ 140/90 mmHg during the health screening or the presence of at least one claim per year for ICD-10-CM codes I10-13, I15, and a prescription for an antihypertensive agent).

### Assessment of NAFLD and pancreatic cancer

NAFLD was assessed using fatty liver index, one of the best-validated fatty-liver-prediction models^10,26^. The 2016 European guidelines recommend non-invasive biomarkers as the preferred diagnostic tools for hepatic steatosis in large-scale population-based studies, as the availability and cost of liver biopsies and imaging substantially impact study feasibility^10^. The fatty liver index, ranging from zero to 100, was calculated as follows; (e^0.953 × Ln(triglyceride) + 0.139 × BMI + 0.718 × Ln (GGT) + 0.053 × waist circumference ± 15.745^)/ (1 + e^0.953 × Ln (triglyceride) + 0.139 × BMI + 0.718 × Ln (GGT) + 0.053 × waist circumference ± 15.745^) × 100^26^. The participants were then categorized into three groups based on criteria used in previous studies: ≥ 60, NAFLD; 30–59, intermediate score; and < 30, no NAFLD^18,21,23,26–32^.

The primary outcome was newly diagnosed pancreatic cancer, which was identified based on hospitalization with the ICD-10-CM code for pancreatic cancer (C25) and a reimbursement code for cancer in the national registration data (V193) between January 2009 and December 2017.

### Statistical analysis

Baseline characteristics were analyzed using a one-way analysis of variance to compare continuous variables and χ^2^ tests for categorical variables. The incidence rates of pancreatic cancer were estimated by dividing the number of incident cases per 1000 person-years. We used multivariable Cox proportional hazards regression models to estimate the adjusted hazard ratios (HRs) and 95% confidence intervals (CIs) for the association between NAFLD and pancreatic cancer risk. Model 1 was adjusted for age and sex, and Model 2 was further adjusted for smoking, alcohol consumption, physical activity, income, BMI, diabetes, and pancreatitis. In addition, we conducted a sensitivity analysis using an inverse probability-weighted analysis, a type of propensity score analysis, to control for confounding by observed covariates^33,34^. We performed subgroup analyses and reported *P-*values for interactions. We also calculated *P*-values for linear trends of pancreatic cancer risk across the fatty liver index categories. All statistical tests were two-sided, and significance was set at a *P*-value of < 0.05. All statistical analyses were performed using SAS software (version 9.3; SAS Institute, Cary, NC, USA).

## Results

### Baseline characteristics

During the mean follow-up period of 7.2 years, 10,470 individuals were newly diagnosed with pancreatic cancer. Table 1 shows the baseline characteristics of the study population. The mean age of the participants was 46.7 ± 14.1 years, and 52.1% of the participants were male. Those with NAFLD were more likely to be male, mild drinkers, current smokers, and physically active than those without NAFLD. In addition, those with NAFLD had higher mean values for BMI, fasting glucose, blood pressure, and total cholesterol levels than those without NAFLD (all *P* < 0.001).

**Table 1:** Baseline characteristics of the study population.

	No NAFLD	Intermediate	NAFLD	*P* value
	(n = 5,348,282)	(n = 1,836,233)	(n = 936,159)	
**Demographics**				
Age, mean (SD), years	45.5 (14.4)	50.0 (13.5)	47.4 (12.7)	< 0.001
Age ≥ 65 years, n (%)	620,271 (11.6)	300,616 (16.4)	105,154 (11.2)	< 0.001
Male, n (%)	2,193,432 (41.0)	1,277,293 (69.6)	763,693 (81.6)	< 0.001
Smoking status, n (%)				< 0.001
Non-smoker	3,767,603 (70.5)	922,267 (50.2)	361,094 (38.6)	
Ex-smoker	554,858 (10.4)	343,155 (18.7)	187,297 (20.0)	
Current smoker	1,025,821 (19.2)	570,811 (31.1)	387,768 (41.4)	
Alcohol consumption^a^, n (%)				< 0.001
Non-drinker	3,158,256 (59.1)	893,769 (48.7)	350,940 (37.5)	
Mild drinker	2,190,026 (41.0)	942,464 (51.3)	585,219 (62.5)	
Physical activity, n (%)	2,677,738 (50.1)	969,368 (52.8)	508,817 (54.4)	< 0.001
Lower income, n (%)	1,528,844 (28.6)	440,320 (24.0)	217,984 (23.3)	< 0.001
**Anthropometric and laboratory findings**				
Body mass index, mean (SD), kg/m^2^	22.2 (2.4)	25.5 (2.2)	27.9 (3.0)	< 0.001
Waist circumference, mean (SD), cm	75.5 (7.0)	85.9 (5.4)	92.1 (6.8)	< 0.001
Systolic BP, mean (SD), mmHg	119.1 (14.3)	126.5 (14.3)	129.8 (14.5)	< 0.001
Diastolic BP, mean (SD), mmHg	74.1 (9.5)	78.8 (9.5)	81.4 (9.9)	< 0.001
ALT, median (IQR), IU/L	17 (13–22)	25 (19–35)	36 (25–52)	< 0.001
AST, median (IQR), IU/L	21 (18–25)	24 (20–29)	29 (23–37)	< 0.001
GGT, median (IQR), IU/L	18 (13–24)	34 (24–49)	60 (39–96)	< 0.001
Total cholesterol, mean (SD), mg/dL	189.1 (34.4)	203.9 (36.6)	212.5 (38.8)	< 0.001
Triglycerides, median (IQR), mg/dL	86 (63–117)	153 (116–203)	224 (164–312)	< 0.001
HDL-C, mean (SD), mg/dL	58.0 (17.6)	51.3 (19.8)	48.8 (20.0)	< 0.001
LDL-C, mean (SD), mg/dL	112.0 (31.7)	119.5 (35.0)	114.0 (38.7)	< 0.001
Fasting glucose, mean (SD), mg/dL	93.2 (17.9)	100.7 (25.1)	106.7 (31.4)	< 0.001
**Comorbidities, n (%)**				< 0.001
Diabetes mellitus	255,004 (4.8)	220,559 (12.0)	166,854 (17.8)	
Hypertension	919,994 (17.2)	644,793 (35.1)	407,835 (43.6)	
Dyslipidemia	651,749 (12.2)	465,358 (25.3)	318,416 (34.0)	

Data are presented as the mean (SD), median (IQR), or number (%).

Fatty liver index: ≥ 60, NAFLD; 30–59, intermediate; < 30, no NAFLD.

*ALT* alanine transaminase, *AST* aspartate aminotransferase, *BP* blood pressure, *GGT* gamma-glutamyl transferase, *HDL-C* high-density lipoprotein cholesterol, *IQR* interquartile range, *LDL-C* low-density lipoprotein cholesterol, *NAFLD* non-alcoholic fatty liver disease, *SD* standard deviation.

^a^Individuals who consumed alcohol ≥ 30 g/day were initially excluded.

### Association between NAFLD and risk of pancreatic cancer

Table 2 presents the risk of pancreatic cancer according to NAFLD status. NAFLD was associated with an increased risk of pancreatic cancer compared to no NAFLD after adjusting for age and sex (in Model 1; HR, 1.36; 95% CI 1.29–1.44). The association persisted even after adjusting for age, sex, alcohol consumption, smoking, physical activity, income, BMI, diabetes, and pancreatitis in Model 2 (hazard ratio [HR], 1.17; 95% CI 1.09–1.26). Intermediate fatty liver index was also significantly associated with an increased risk of pancreatic cancer in all models (HR; 95% CIs: [1.19; 1.14–1.24] and [1.10; 1.05–1.16] for Models 1 and 2, respectively). The adjusted HRs of pancreatic cancer tended to increase progressively with increasing fatty liver index (*P* for trend < 0.001). Even after applying inverse probability weights, a significant association between NAFLD and pancreatic cancer was consistently observed (Supplementary Table 1).

**Table 2:** Association between non-alcoholic fatty liver disease and the risk of pancreatic cancer.

	Event, n	Duration (person-years)	IR^a^	HR (95% CI)		*P* for trend
				Model 1	Model 2	
No NAFLD	5,760	38,963,103	1.48	1 [Reference]	1 [Reference]	< 0.001
Intermediate	3,186	13,343,600	2.39	1.19 (1.14–1.24)	1.10 (1.05–1.16)	
NAFLD	1,524	6,785,211	2.25	1.36 (1.29–1.44)	1.17 (1.09–1.26)	

Fatty liver index: ≥ 60, NAFLD; 30–59, intermediate; < 30, no NAFLD.

Model 1 was adjusted for age and sex.

Model 2 was adjusted for age, sex, smoking status, alcohol consumption, physical activity, income, diabetes, pancreatitis, and body mass index.

*CI* confidential interval, *HR* hazard ratio, *NAFLD* non-alcoholic fatty liver disease.

^a^IR, the incidence rate per 10,000 person-years.

### Subgroup analyses

**Figure 2:**
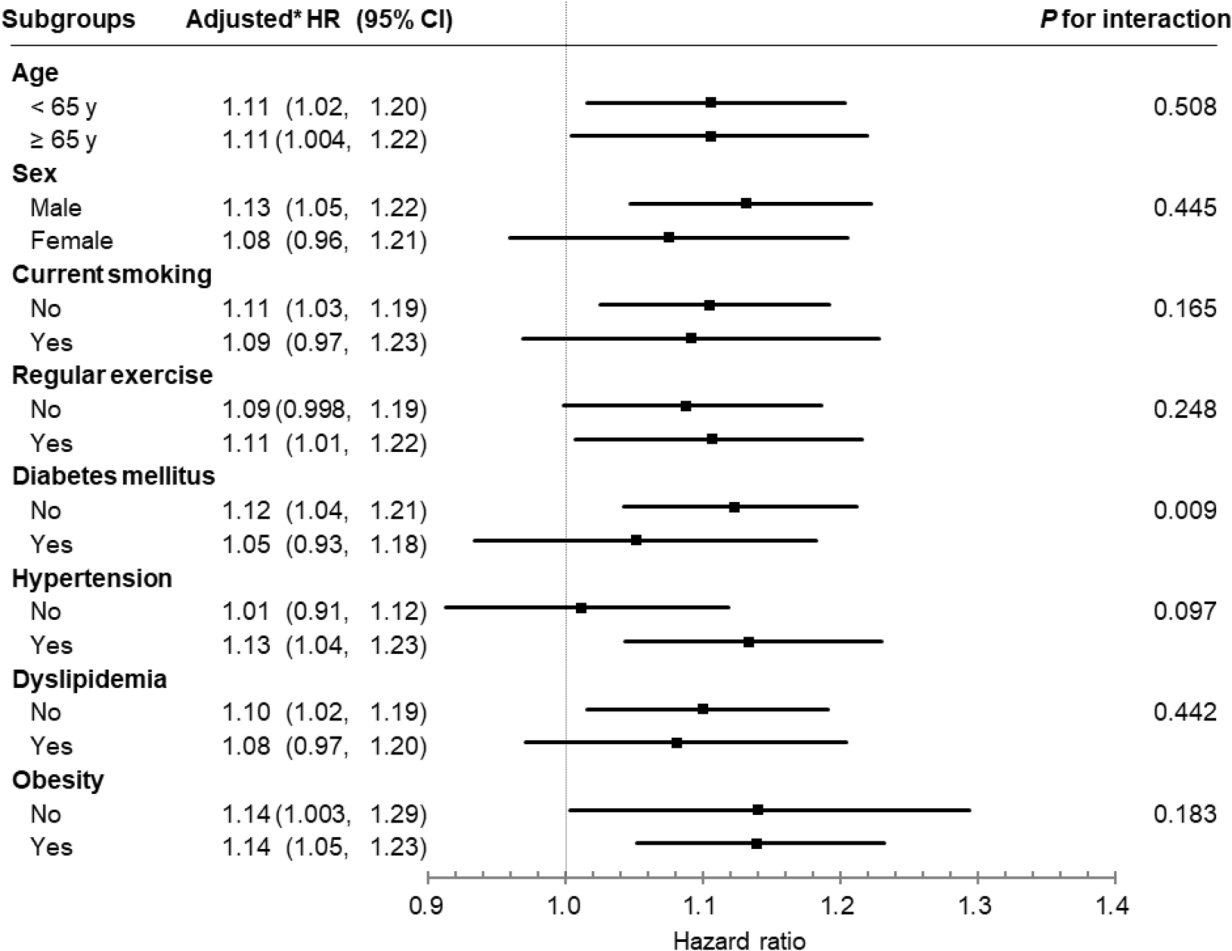
Association between non-alcoholic fatty liver disease and the risk of pancreatic cancer by subgroup. Forrest plots of hazard ratios (HRs) and 95% confidential intervals (CIs) adjusted for age, sex, smoking, alcohol consumption, physical activity, income level, body mass index, diabetes, and pancreatitis according to subgroups.

Figure 2 presents the adjusted HRs of pancreatic cancer and *P* for interactions after adjusting for multiple confounders in each subgroup. There were no significant interactions between NAFLD and stratified variables, except for diabetes (*P* = 0.009). The association between NAFLD and pancreatic cancer risk did not differ according to age, sex, smoking, physical activity, hypertension, dyslipidemia, or obesity (all *P* > 0.05; Fig. 2). The association between NAFLD and the risk of pancreatic cancer was significant in both obese (HR, 1.14; 95% CI 1.05–1.23) and non-obese groups (HR, 1.14; 95% CI 1.003–1.29).

### Combined effects of NAFLD and smoking on pancreatic cancer risk

Table 3 shows the risk of pancreatic cancer according to the combination of NAFLD and smoking after adjusting for multiple confounders. Compared to non-smokers without NAFLD, non-smokers with NAFLD and smokers without NAFLD showed an increased risk of pancreatic cancer (HR, 1.12; 95% CI 1.04–1.21; and HR, 1.38; 95% CI 1.29–1.47, respectively). The combined effects of NAFLD and smoking further increased the risk of pancreatic cancer by 42% (HR 1.42; 95% CI 1.28–1.58).

**Table 3:** Combined effects of non-alcoholic fatty liver disease and smoking on the risk of pancreatic cancer.

Smoking status	NAFLD status	Event, n	Duration (person-years)	IR^a^	Adjusted HR (95% CI)
No smoking	No NAFLD	6960	40,736,870	1.71	1 [Reference]
	NAFLD	1041	3,978,411	2.62	1.12 (1.04–1.21)
Smoking	No NAFLD	1986	11,569,832	1.72	1.38 (1.29–1.47)
	NAFLD	483	2,806,800	1.72	1.42 (1.28–1.58)

HRs and 95% CIs were adjusted for age, sex, smoking status, alcohol consumption, physical activity, income, diabetes, pancreatitis, and body mass index.

*CI* confidential interval, *HR* hazard ratio, *NAFLD* non-alcoholic fatty liver disease.

^a^IR, the incidence rate per 10,000 person-years.

## Discussion

This nationwide cohort study, including over 8 million individuals, demonstrated that NAFLD was independently associated with an increased risk of pancreatic cancer after adjusting for potential confounders. The significant association between NAFLD and the risk of pancreatic cancer was consistent in both the obese and non-obese groups. The risk of pancreatic cancer tended to increase as the fatty liver index increased. To the best of our knowledge, the present study is the first nationwide cohort study to demonstrate an association between NAFLD and the risk of pancreatic cancer in the general population, regardless of obesity.

Limited data are available on the association between NAFLD and pancreatic cancer. Only a few hospital-based studies have examined the association between NAFLD and pancreatic cancer risk. However, previous studies showed inconsistent results with positive^11–14^ or null associations^15^. Furthermore, previous studies have possible limitations (Supplemental Table 2). First, all previous studies were performed in hospitals, even though NAFLD is generally asymptomatic; thus, these studies may not include a substantial proportion of asymptomatic patients with NAFLD and included a greater proportion of NAFLD patients with other confounding comorbidities^11–15^. Therefore, these studies might have a selection bias and were not representative of the general population. Second, previous studies included a limited number of pancreatic cancer cases. These studies analyzed pancreatic cancer risk using sample sizes ≤ 188 cases, which can also lead to undercoverage bias. Third, previous studies did not consider significant confounders related to pancreatic cancer, such as pancreatitis^11–15^, BMI^11–15^, and smoking^11,12,14^. The results of previous studies are summarized in Supplemental Table 2. Recently, beyond the concept of NAFLD, evidence for an association between metabolic dysfunction–associated fatty liver disease (MAFLD) and pancreatic cancer risk has been suggested^35^. MAFLD is defined as a fatty liver disease with metabolic abnormalities, such as overweight, diabetes, and metabolic syndrome.

Several potential biological mechanisms underlie the increased pancreatic cancer risk in patients with fatty liver disease. First, systemic release of proinflammatory cytokines in hepatic steatosis can induce chronic, low-grade systemic inflammation^36,37^. Inflammation has diverse tumor-promoting effects, such as cell proliferation and inhibition of adaptive immunity, in cancers, including pancreatic cancer^38,39^. Several studies have reported that diverse inflammatory signaling pathways, such as NF-κB, the IL-6-STAT3 axis, and TGF-β, promote the carcinogenesis of pancreatic cancer^40,41^. Second, the altered microbiome in patients with hepatic steatosis can increase pancreatic cancer risk^42,43^. Recently, the role of gut microbiota in the pathophysiology of various human diseases, such as metabolic diseases, inflammatory diseases, and cancers, has been revealed. Gut dysbiosis is significantly involved in hepatic steatosis progression and carcinogenesis in different cancers, including pancreatic cancer, via the gut-liver axis^42,43^. Third, insulin resistance is a key factor in the pathophysiology of hepatic steatosis. Insulin resistance increases cell proliferation, cellular mobility, angiogenesis, and damage to DNA molecules by active forms of oxygen^37,44^. Last, insulin-like growth factor-1, which is associated with hepatic steatosis, inhibits apoptosis and promotes progression through the cell cycle, and may play a role in pancreatic carcinogenesis.

It can be argued that the association between NAFLD and pancreatic cancer risk may be due to shared risk factors, such as obesity. However, regardless of obesity, the significant association between NAFLD and pancreatic cancer risk was consistent after adjusting for potential confounders. The current concept of “non-obese fatty liver disease” or “lean NAFLD” supports the hypothesis of an independent effect of NAFLD on the development of pancreatic cancer in the absence of obesity^45–47^.

This study also showed that a combination of NAFLD and smoking further increased the risk of pancreatic cancer. Our findings demonstrate that the combined effect of these two modifiable risk factors has significant clinical implications for lowering the risk of pancreatic cancer.

This cohort study has several strengths. First, the KNHIS data covers the entire Korean population, and all medical records are tracked in the database. Second, this cohort study used systematically and longitudinally collected measurements and clinical data at an individual level prior to the incidence of pancreatic cancer in a vast population of over 8 million people. The recall bias was considered to be minimal in our study. Third, to achieve a high diagnostic accuracy for pancreatic cancer, we used both diagnostic and special reimbursement codes (C and V codes). Last, we obtained both health screening data and KNHIS claim data; hence, we could adjust for confounding factors for pancreatic cancer, including pancreatitis, BMI, diabetes, physical activity, and smoking.

Our study has several limitations. First, we tried to minimize reverse causality by excluding those who had a cancer diagnosis before or were diagnosed with pancreatic cancer or died within 1 year after cohort entry. Nonetheless, there may be a possibility of reverse causality. Second, the information on the pathological subtype of pancreatic cancer was not obtained. However, pancreatic adenocarcinoma accounts for approximately 90% of all pancreatic cancers, and pancreatic neuroendocrine tumors are rare, accounting for less than 5% of all cases^32^. Last, although we adjusted for multiple confounders, including pancreatitis, smoking, and BMI, the possibility of residual confounding cannot be excluded.

In conclusion, this nationwide cohort study demonstrated that NAFLD was significantly associated with an increased risk of pancreatic cancer in the general Korean population. The association was significant regardless of obesity status. In addition, the combined effects of NAFLD and smoking further increased the risk of pancreatic cancer. Our findings suggest that NAFLD is a modifiable risk factor for pancreatic cancer. Further studies are needed to investigate the effects of NAFLD management on reducing the risk of pancreatic cancer.

## Supplementary Information

Supplementary Information.

## Data Availability

The Korean National Health Insurance Service (KNHIS) permits researchers to access the KNHIS data after reviewing the research topic. Requests for access to the data can be made on the KNHIS website.
