# Supplementary material for: Increased risk of pancreatic cancer in individuals with non-alcoholic fatty liver disease
Source: Sci Rep. 2022 Jun 23;12:10681. doi: 10.1038/s41598-022-14856-w (PMC9226051; doi:10.1038/s41598-022-14856-w)
Supplement: Supplementary file 1 — Supplementary Information. [file 41598_2022_14856_MOESM1_ESM.pdf]

Supporting Information

Increased risk of pancreatic cancer in individuals with non-alcoholic fatty liver disease

Joo-Hyun Park, Jung Yong Hong, Kyungdo Han, Wonseok Kang, and Joo Kyung Park

Supplementary Table 1. Association between non-alcoholic fatty liver disease and the risk of pancreatic cancer after application of inverse probability weights

	Event, n	Duration (person-years)	IR[a]	Inverse-weighted and adjusted HR (95% CI)	*P* for trend
No NAFLD	5,760	38,963,103	1.48	1 [Reference]	< 0.001
Intermediate	3,186	13,343,600	2.39	1.13 (1.08-1.19)	
NAFLD	1,524	6,785,211	2.25	1.27 (1.18-1.38)	

[a]IR, the incidence rate per 10,000 person-years

Fatty liver index: ≥60, NAFLD; 30-59, intermediate; <30, no NAFLD.

Inverse-weighted and adjusted HR was adjusted for age, sex, smoking status, alcohol consumption, physical activity, income, diabetes, pancreatitis, and body mass index.

CI, confidential interval; HR, hazard ratio; NAFLD, non-alcoholic fatty liver disease.

Supplementary Table 2. Summary of previous studies and the current study on the association between non-alcoholic fatty liver disease and pancreatic cancer risk

	Year	Country	No. of total cases	No. of PaC cases	F/U (y)	Data source	Definition of NAFLD	OR/HR (95% CI)
**Sørensen**[1]	2003	Denmark	7,326	16	6.2	One hospital	ICD-8-CM code	3.0 (1.3-5.8)
**Chang**[2]	2018	Taiwan	557	143	N/A	One hospital	Computed tomography	2.63 (1.24-5.58)
**Kim**[3]	2017	South Korea	25,947	24	7.5	One hospital	Sonography	1.16 (0.51-2.65)
**Allen**[4]	2019	USA	19,163	72	21	65 health care providers in a county	ICD-10-CM code	2.0 (1.2-3.3)
**Simon**[5]	2021	Sweden	48,799	188	13.8	Twenty-eight Swedish hospitals	Biopsy	2.15 (1.40-3.30)
**Current study**	2021	South Korea	8,120,674	10,470	7.2	General population who underwent a national health screening	Fatty liver index	1.17 (1.09-1.26)

CI, confidence interval; F/U, follow-up; HR, hazard ratio; ICD-8-CM, International Classification of Disease, Eighth Revision, Clinical Modification; NAFLD, non-alcoholic fatty liver disease; OR, odds ratio; PaC, pancreatic cancer; y, years.

References

1. Sørensen, H. T. *et al.* Risk of cancer in patients hospitalized with fatty liver: a Danish cohort study. *J Clin Gastroenterol* **36**, 356-359. https://doi.org/10.1097/00004836-200304000-00015 (2003).
2. Chang, C. F. *et al.* Exploring the relationship between nonalcoholic fatty liver disease and pancreatic cancer by computed tomographic survey. *Intern Emerg Med* **13**, 191-197. https://doi.org/10.1007/s11739-017-1774-x (2018).
3. Kim, G. A. *et al.* Association between non-alcoholic fatty liver disease and cancer incidence rate. *Journal of Hepatology* **68**, 140-146. https://doi.org/10.1016/j.jhep.2017.09.012 (2018).
4. Allen, A. M., Hicks, S. B., Mara, K. C., Larson, J. J. & Therneau, T. M. The risk of incident extrahepatic cancers is higher in non-alcoholic fatty liver disease than obesity - A longitudinal cohort study. *J Hepatol* **71**, 1229-1236. https://doi.org/10.1016/j.jhep.2019.08.018 (2019).
5. Simon, T. G. *et al.* Cancer Risk in Patients With Biopsy-Confirmed Nonalcoholic Fatty Liver Disease: A Population-Based Cohort Study. *Hepatology* **74**, 2410-2423. https://doi.org/10.1002/hep.31845 (2021).
